# The contrast-enhanced Doppler ultrasound with perfluorocarbon exposed sonicated albumin does not improve the diagnosis of renal artery stenosis compared with angiography

**DOI:** 10.1186/1477-5751-3-3

**Published:** 2004-09-20

**Authors:** Odila UN Teixeira, Luiz A Bortolotto, Hélio Bernardes Silva

**Affiliations:** 1 Hypertension Unity, Heart Institute (InCor), São Paulo Medical School, Brazil

**Keywords:** renal artery stenosis, ultrasound, PESDA, angiography, hypertension

## Abstract

There are no studies investigating the effect of the contrast infusion on the sensitivity and specificity of the main Doppler criteria of renal artery stenosis (RAS). Our aim was to evaluate the accuracy of these Doppler criteria prior to and following the intravenous administration of perfluorocarbon exposed sonicated albumin (PESDA) in patients suspected of having RAS. Thirty consecutive hypertensive patients (13 males, mean age of 57 ± 10 years) suspected of having RAS by clinical clues, were submitted to ultrasonography (US) of renal arteries before and after enhancement using continuous infusion of PESDA. All patients underwent angiography, and haemodynamically significant RAS was considered when ≥50%. At angiography, it was detected RAS ≥50% in 18 patients, 5 with bilateral stenosis. After contrast, the examination time was slightly reduced by approximately 20%. In non-enhanced US the sensitivity was better when based on resistance index (82.9%) while the specificity was better when based on renal aortic ratio (89.2%). The predictive positive value was stable for all indexes (74.0%–88.0%) while negative predictive value was low (44%–51%). The specificity and positive predictive value based on renal aortic ratio increased after PESDA injection respectively, from 89 to 97.3% and from 88 to 95%. In hypertensives suspected to have RAS the sensitivity and specificity of Duplex US is dependent of the criterion evaluated. Enhancement with continuous infusion of PESDA improves only the specificity based on renal aortic ratio but do not modify the sensitivity of any index.

## Introduction

Renal artery stenosis is the most frequent cause of secondary hypertension [1] which is potentially treatable with angioplasty, endovascular stent placement or surgical revascularization [2,3]. The angiography remains the gold standard, however, is invasive, expensive, and potentially harmful specially in patients with compromised renal function or diabetes [4]. Over the past few years, there has been extensive research for a reliable, noninvasive, and nonionizing imaging method to screen for renal artery stenosis (RAS) [5]. Magnetic resonance (MR) angiography, captopril renography and duplex ultrasonography have all been assessed for this purpose [6,7]. Duplex ultrasonography (US) is safe and widely available, but its use as a screening tool of renal artery stenosis does not have universal acceptance because of the lack of standardization in examination protocols and diagnostic criteria, as well as the wide differences in reported accuracy among different laboratories [8,9]. In addition, despite the use of color Doppler and other technological improvements, the localization of the main renal arteries deep within the abdomen has rendered direct visualization of these vessels difficult [10]. There is a 10 to 20 percent rate of failure due to the operator's inexperience, the presence of obesity, overlying bowel gas or respiratory motion [11]. 

The proposed criteria for the detection of renal artery stenosis by direct Doppler include an increased peak systolic velocity, an increase in renal aortic ratio and also an increased resistance index [12]. The mean sensitivity and specificity of the Duplex US based on these criteria varies, respectively, from 10 to 93% and from 37% to 100%, according to different reports [8-10,12-20]. There are few reports comparing these criteria for the detection of RAS [15].

Recently, the use of microbubble echo-enhancing agents in combination with harmonic Doppler imaging has been proposed to improve Doppler signal intensity in multiple vascular sites [21]. Thus, it would be expected to improve the operator's ability to visualize the renal arteries, and to significantly reduce the number of equivocal examinations [11,21-23]. In addition, contrast-enhanced harmonic Doppler US can currently provide objective functional assessment of RAS through analysis of time-intensity renal enhancement curve [22]. MISSOURIS et al [23] have reported data of microbubble Levovist^®^ echo-enhancing ultrasonography in hypertensives with renal artery stenosis. They demonstrated a sensitivity of 85% and a specificity of 79% without contrast and a sensitivity of 94% and a specificity of 88% with contrast, besides an important reduction in the time of procedure. The echo-enhancing agent PESDA (perfluorocarbon exposed sonicated albumin) is a second-generation agent, containing high molecular weight gas, whose use results in higher stability and better reflections of Doppler signs [24]. The PESDA is broadly used in echocardiography [24], but till date there is no studies using PESDA contrast in echo-enhanced US of renal arteries. Also there are no studies that investigate the effect of the contrast infusion on the sensitivity and specificity of the different Doppler criterion mentioned above. 

The purpose of our study was to evaluate the accuracy of the main color Doppler criteria of the renal arteries prior to and following the intravenous administration of PESDA in patients suspected of having renal arterial stenosis. These results were compared with those from conventional angiography, which was regarded as the standard of reference. As a secondary objective, the feasibility, time of examination and safety of US with PESDA infusion was analyzed. 

## Methods

### Study Population

Thirty patients (13 males/ 17 females), with a mean age of 57 ± 12 years (range 16–77 years) were enrolled in the study, that was performed at Heart Institute of São Paulo University. The only inclusion criterion was a clinical suspicion of renal arterial stenosis that required conventional or digital subtraction angiography for diagnosis. The suspicion of renovascular hypertension was based on the presence of one or more of the following clinical features: resistant hypertension, progressive renal failure with no recognized cause, atherosclerotic disease in other circulatory site (coronary, peripheral or cerebrovascular disease), renal failure induced by ACE inhibitors; renal asymmetry; retinopathy grade lll or lV (Keith Wagener) with diastolic blood pressure over 125 mmHg [1]. Exclusion criteria were as follows: patients with a renal transplant; patients who had a renal arterial stent and those referred because they were suspected of having renal arterial restenosis; patients who received any iodinated agent in the previous 24 hours, and patients with acute myocardial infarction or stroke. All patients underwent conventional angiography or digital subtraction angiography. 

The patients were assigned randomly in a 2-steps imaging protocol: 1) acquisition of a baseline non-enhanced Doppler ultrasound study by a Sequoia Echography System (Acuson, Siemens, Mountain View, CA, USA); 2) a continuos infusion of contrast PESDA, 0,1 ml per kg of weight in a rate of infusion of 2 ml per minute; c) after infusion, we adjust the gain according to the observed gain intensity increase for optimal filling of the vessel lumen in color mode and delineation of the spectrum envelope in duplex mode. The ultrasound scans were done in different positions to evaluate all segments of the main renal arteries: a) supine position to visualize the origin and proximal portion, b) epigastric transverse scans of the aorta to identify the right artery (anterolateral) and left artery (posterolateral), c) the sagital or coronal scan from a flank approach to identify the medium and distal portions of the arteries [[20],26,27]. Peak systolic (PSV) and diastolic (PDV) velocities of the aorta and renal arteries, and calculation of resistance index (RI), pulsatility index (PI) and renal aortic ratio (RAR) were obtained in all segments of renal arteries [[20],26-27].

The following spectral Doppler diagnostic criterion for renal arterial stenosis were used: a) PSV > 150 cm/s [26-27]; b) RAR > 3.0 [18]; c) RI > 0,80 [17]. The same examiner performed all examinations and the confirmation of the presence of stenosis was done in consensus with another examiner, both of them blinded to the results of the angiography.

We also collected clinical data, including number of drugs, serum creatinine and values of blood pressure in baseline conditions. 

The results of PESDA-enhanced and non-enhanced ultrasound examinations were compared with those from intraarterial angiography. A hemodynamically significant stenosis was defined as diameter reduction of 50% or more at angiography, because it has been widely used in the recent literature [13]. The radiologist and clinician interpreting the study were blinded to the Doppler examination results. 

Secondary efficacy variables included the duration of each Doppler examination, the detection of supernumerary arteries and adverse effects. 

The study was approved according to local legal requirements and informed consent was obtained before ultrasound examination from all patients.

## Results

### Patient Characteristics 

All patients underwent digital subtraction angiography and non-enhanced US. One patient did not receive the infusion of contrast, because the venous access was not possible. Renal arterial stenosis of 50% or greater was detected at angiography in 18 (60%) patients, 5 of whom had bilateral stenosis. Renal arterial stenosis was excluded in 12 patients. Thus, stenosis by angiography was detected in 23 arteries, while 37 arteries did not present. The clinical and demographic data of patients according to the presence of stenosis are presented in the Table 1. The patients with stenosis were older than patients with no stenosis (p = 0.013), while we did not observe differences in the other demographic and clinical data. 

**Table 1:** Demographic and clinical data of 30 patients according to the presence of RAS at angiography

	Patients with no stenosis (n = 12)	Patients with stenosis (n = 18)	p
Age (years)	43 ± 15	57 ± 14	0,013*
BMI	25,5 ± 5,2	26,5 ± 3,4	0,528
SP (mmHg)	158 ± 29	162 ± 26	0,738
DP (mmHg)	97 ± 15	96 ± 16	0,900
HR	74 ± 12	74 ± 11	0,866
Cr	1,21 ± 0,51	2,01 ± 1,50	0,088

BMI: corporeal mass index SP: systolic pressure DP: diastolic pressure; HR: heart rate Cr: serum creatinine (normal < 1,50 mg/dl); RAS = renal artery stenosis

### Feasibility

Overall, all patients had the renal arteries assessable with non-enhanced US or after injection of PESDA, although in one patient with a high body mass index, the assessment was better after contrast. Despite an expectation of at least 20% accessory arteries, we did not find any in our population.

### Accuracy

As stenosis is mostly in ostium and proximal portion of the arteries, we considered for diagnosis of renal artery stenosis the indexes obtained by echo-Doppler in these arterial segments. The mean values of PSV, PDV, RAR, RI and PI in these segments are showed in the tables 2 and 3. In the ostium, the values of PSV and RAR were higher in arteries with stenosis, in either enhanced or non-enhanced US. In the proximal segment, only RAR values were higher in arteries with stenosis, in both enhanced and non-enhanced US. 

**Table 2:** Mean values of Doppler indexes obtained with non-enhanced or enhanced with PESDA ultrassonography in the ostium of renal arteries

	**Non-enhanced**		**Enhanced**		**p**	
	**Arteries without stenosis n = 37**	**Arteries with stenosis n = 23**	**Arteries without stenosis n = 37**	**Arteries with stenosis n = 23**	**Stenosis vs no stenosis**	**Enhanced vs no enhanced**

PSV (cm/s)	1,49 ± 0,76	2,26 ± 1,15	1,49 ± 0,65	2,01 ± 1,27	**0,001**	0,975
PDV (cm/s)	0,31 ± 0,25	0,42 ± 0,34	0,32 ± 0,19	0,37 ± 0,38	0,229	0,596
RRA	1,43 ± 0,67	2,36 ± 1,34	1,19 ± 0,54	2,19 ± 1,45	**<0,001**	0,145
RI	0,77 ± 0,20	0,84 ± 0,12	0,79 ± 0,11	0,76 ± 0,27	0,601	0,291
PI	1,83 ± 0,90	2,23 ± 0,97	1,97 ± 0,77	1,97 ± 0,99	0,332	0,588

PSV: peak systolic velocity; PDV: peak diastolic velocity; RAA: renal/aortic ratio RI: resistance index; PI: pulsatility index; US = ultrasonography

**Table 3:** Mean values of Doppler indexes obtained with non-enhanced or enhanced with PESDA ultrassonography in the proximal portion of renal arteries

	**Non-enhanced**		**Enhanced**		**p**	
	**Arteries without stenosis n = 37**	**Arteries with stenosis n = 23**	**Arteries without stenosis n = 37**	**Arteries with stenosis n = 23**	**Stenosis vs no stenosis**	**Enhanced vs no enhanced**

PSV (cm/s)	1,56 ± 0,79	2,12+/-1,22	1,68+/-0,86	2,01+/-1,27	0,059	0,973
PDV (cm/s)	0,36+/-0,22	0,38+/-0,31	0,33+/-0,21	0,38+/-0,36	0,589	0,618
RAR	1,54+/-0,84	2,16+/-1,25	1,30+/-0,60	1,98+/-1,36	**0,008**	0,103
RI	0,76+/-0,11	0,83+/-0,11	0,78+/-0,13	0,76+/-0,26	0,193	0,793
PI	1,74+/-0,68	2,31+/-0,91	2,06+/-1,01	2,05+/-1,07	0,526	0,375

PSV: peak systolic velocity; PDV: peak diastolic velocity; RAA: renal/aortic ratio RI: resistance index; PI: pulsatility index; US = ultrasonography

The sensitivity, specificity, positive predictive value and negative predictive value of each Doppler index for the detection of RAS (Table 4) were calculated based on the values standardized in the literature. In terms of renal arteries, we observe that the sensitivity and specificity depend on the index used. Thus, in non-enhanced US the sensitivity was better when based on RI (82.9%) while the specificity was better when based on RAR (89.2%). The PPV was stable for all indexes (74.0%–88.0%) while NPV was low (44%–51%). The specificity and PPV based on RAR increased after PESDA injection respectively, to 97.3% and 95%. 

**Table 4:** Sensitivity and specificity of non-enhanced and enhanced Doppler US for the detection of RAS in 60 arteries

	a) Non-enhanced			
	Sensitivity	Specificity	PPV	NPV

RRA (<3,0)	56,2%	89,2%	88%	44%
PSV (<150)	69,7%	64,9%	75%	45%
RI (<0,80)	82,9%	56,8%	74%	51%

	b) Enhanced			

	Sensitivity	Specificity	PPV	NPV

RRA (<3,0)	33,3%	97,3%	95%	40%
PSV (<150)	61,9%	64,9%	72%	42%
RI (<0,80)	76,2%	43,2%	66%	42%

RRA: ratio renal/aortic PSV: peak sistolyc velocity(cm/s) RI: resistive index PPV = positive predictive value, NPV = negative predictive value RAS = Renal artery stenosis; US = ultrasonography

The receiver operating characteristic curves for each Dopplerdiagnostic criterion showed that the area under the receiver operating characteristic curve for resistance index was greater than the area under the curve for peak systolic velocity and renal aortic ratio. For renal aortic ratio, the cutoff point that provided the best accuracy, 2.7 gave a specificity of 96% but a low sensitivity (60%). For peak systolic velocity, no precise cutoff point could be identified between arteries with stenosis and those without stenosis. For resistance index, a threshold of 0.8 led to a sensitivity of 70% and a low specificity of 56.8%. 

### Secondary variables and safety

The median examination time was 35 minutes for enhanced Doppler US and 29 minutes for non-enhanced Doppler US, i.e., a small but significant reduction of 17% (p = 0.03). Only one patient presented adverse events to be potentially related to the injection of PESDA, including sensation of coldness, palpitation and dyspnea. There was no severe adverse event. 

## Discussion

Although the technique of renal arterial US scanning has been well established for years, a lot of difficulties in reliably identifying main and accessory renal arteries remain [8-10,25]. Most of these dificulties are related to the patient obesity, the presence of bowel gas, excessive respiratory movement, and the depth and tortuosity of the renal arteries [8,16]. The time expended in the examination can be too long as almost 60 minutes [19], and failure of technique varies from 9 to 25%. In our study, non-enhanced Doppler US showed a feasibility rate of 100%, similar to some single centers, but higher than the majority of studies using this technique (58–90%) [11]. Indeed, two recently published studies reported feasibility not exceeding 11% and 12% [22,23]. One of the reasons of our high rate of feasibility probably is related to the quality of the machine, which allowed a scan imaging with an excellent definition. To our knowledge, the present study is the first randomized study in a selected group of hypertensive patients in which renal arterial color Doppler flow US with and without a continuous infusion of PESDA was compared against the reference standard of angiography. The infusion of PESDA did not alter the feasibility that remains 100%. In a multicentric study [11] using Levovist as the US contrast, the infusion increased by 20% the number of patients in whom renal arteries could be evaluated, including difficult cases such as those involving patients who are obese and patients with impaired renal function. However, some centers participating of the study also presented a feasibility of 100% and the Levovist infusion did not interfere in the results. In our study only one obese patient had a better visualization of renal artery after contrast infusion, and in all patients with renal failure, the non-enhanced US was able to localize renal arteries. 

The most important conclusion from this study is that both sensitivity and specificity of Doppler US of renal arteries are strongly dependent on the criterion used, and the infusion of PESDA contrast seems not to improve it significantly, although we observe a slight increase in specificity. Thus, the best sensitivity was obtained when based on resistance index <0.8 (82.9%) but at expense of a low specificity (56.8%). On the other hand, the best specificity was obtained with renal aortic ratio >3 (89.2%), but the sensitivity was low (56.2%). In addition, the sensitivity and specificity for a peak systolic velocity of 1.5 m/sec showed intermediate values, respectively, 61.9% and 64.9%. An analysis of previously published studies [8-10,12,15,16,20] based on non enhanced Doppler evaluation of the renal artery clearly shows that the diagnostic criteria and respective threshold values fluctuate from one report to the other. Miralles et al [15] reported a sensitivity of 87.3% and a specificity of 91.5% for a higher peak systolic velocity (>1.98 m/sec) and a higher renal aortic ratio (>3.3), while Olin et al [12] reported a sensitivity of 98% and specificity of 98% for a quite similar criteria. Helenon et al [10] quoted a sensitivity of 89% and a specificity of 99% with use of a peak systolic velocity cutoff point similar to our study (1.5 m/sec) but taking into account the presence of poststenotic turbulence and not renal aortic ratio. Moreover, in the multicentric study cited above comparing non enhanced and enhanced Doppler US [11], renal aortic ratio was more accurate than peak systolic velocity in the diagnosis of a renal arterial stenosis greater than 50%, but it was difficult to determine a precise cutoff point. In the same study, the authors demonstrated, in terms of patients, a sensitivity of 80.0% and a specificity of 80.8%, but according to renal arteries the sensitivity was lower (66.7%) and the specificity was higher (90.4%). These latter results were quite similar to our results based on RAR criteria, also evaluated according to renal arteries. These facts, determination of accuracy in terms of renal arteries and not in terms of patients, can explain in part the differences encountered between our conclusions and those from the studies mentioned above. 

The continuous infusion of PESDA contrast increased moderately the specificity for renal aortic ratio criteria from 89.2% to 97.3% but at the expense of a significant decrease of sensitivity from 56.2% to 33.3%. For the another criteria, peak systolic velocity and resistance index the infusion of PESDA decreased mildly or did not affect the sensitivity and specificity. MISSOURIS et al [23] have reported an increase of sensitivity from 85% to 94% and of specificity from 79% to 88% after injection of microbubble Levovist^®^ in hypertensives with renal artery stenosis. In a more recent multicentric study the contrast Levovist did not affect either sensitivity or specificity: sensitivity was 80.0%–83.7%, whereas specificity moderately increased from 80.8% to 83.6% or 86.2%, depending on the subgroups of comparable patients. In two single-center studies in which the value of Doppler US examination after intravenous injection of contrast agents for the diagnosis of renal arterial was also evaluated it was demonstrated an improvement in sensitivity, which increased from 83% to 95% in one study and from 75% to 100% in the other [22,23]. However, both of these studies were based on a limited number of patients with a very low feasibility rate of 11% and 12% at baseline examination, respectively. In addition, Melany et al [22] reported that contrast Levovist injection did not improve specificity, as we also demonstrated with PESDA infusion. 

There is no consensus whether contrast agent injection potentially reduces examination duration. In our study, we found a significant reduction of mean examination time after contrast infusion (17%). In other study, it was reported that the use of Levovist dramatically reduced the mean examination time from 24.5 minutes to 13.5 minutes [23]. This advantage could be of potential economic interest, but subsequent studies have to confirm more significant differences. 

PESDA was well tolerated and did not compromise the safety of US. This excellent patient tolerance has already been demonstrated in stress echocardiograph studies that used PESDA as contrast agent [24]. 

The small number of patients impose some limitations to the present study, However, the high prevalence of renal artery stenosis in this selected group of hypertensives counterbalance this limitation. 

In conclusion, the detection of renal artery stenosis by Doppler US depends on the criteria used and infusion of PESDA contrast seems not to improve the accuracy, despite a reduction in the examination duration and **an **increase **in **specificity based **on **one Doppler criterion. Also, the feasibility of US is dependent of the quality of the machine, and the infusion of contrast does not add advantages if the performance of the US machine is excellent. However, it remains unknown if the PESDA infusion can improve feasibility if the machine does not have a good imaging quality. So, there is a need for establishing a consensus opinion regarding Doppler useful criteria and thresholds for the diagnosis of renal arterial stenosis, regardless of the US equipment used or infusion of ultrasonographic contrast.
